# Prenatal dietary supplements influence the infant airway microbiota in a randomized factorial clinical trial

**DOI:** 10.1038/s41467-020-14308-x

**Published:** 2020-01-22

**Authors:** Mathis H. Hjelmsø, Shiraz A. Shah, Jonathan Thorsen, Morten Rasmussen, Gisle Vestergaard, Martin S. Mortensen, Asker Brejnrod, Susanne Brix, Bo Chawes, Klaus Bønnelykke, Søren J. Sørensen, Jakob Stokholm, Hans Bisgaard

**Affiliations:** 1 COPSAC, Copenhagen Prospective Studies on Asthma in Childhood, Herlev and Gentofte Hospital, University of Copenhagen, 2100 Copenhagen, Denmark; 2 Section of Chemometrics and Analytical Technologies, Department of Food Science, University of Copenhagen, 1958 Frederiksberg C, Denmark; 3 Section of Microbiology, Department of Biology, University of Copenhagen, 2100 Copenhagen, Denmark; 4 Department of Biotechnology and Biomedicine, Technical University of Denmark, Soltofts Plads, 2800 Kongens Lyngby, Denmark

**Keywords:** Microbial communities, Inflammatory diseases, Asthma

## Abstract

Maternal dietary interventions during pregnancy with fish oil and high dose vitamin D have been shown to reduce the incidence of asthma and wheeze in offspring, potentially through microbial effects in pregnancy or early childhood. Here we analyze the bacterial compositions in longitudinal samples from 695 pregnant women and their children according to intervention group in a nested, factorial, double-blind, placebo-controlled, randomized trial of n-3 long-chain fatty acids and vitamin D supplementation. The dietary interventions affect the infant airways, but not the infant fecal or maternal vaginal microbiota. Changes in overall beta diversity are observed, which in turn associates with a change in immune mediator profile. In addition, airway microbial maturation and the relative abundance of specific bacterial genera are altered. Furthermore, mediation analysis reveals the changed airway microbiota to be a minor and non-significant mediator of the protective effect of the dietary interventions on risk of asthma. Our results demonstrate the potential of prenatal dietary supplements as manipulators of the early airway bacterial colonization.

## Abstract (web summary)

Here, the authors present the results of a mother–child cohort randomized clinical trial of n-3 LCPUFA and vitamin D maternal supplementation, finding an association between supplement-induced microbiota changes in infant airways at age 1-month but not the infant fecal or maternal vaginal microbiome.

## Introduction

Asthma and other chronic inflammatory diseases are a growing burden in the developed world causing substantial morbidity and mortality^1^. There is substantial evidence pointing to the early life as a critical window for disease development, beginning already in the intrauterine environment^2^. This concept was proven in recent clinical trials of dietary interventions with n-3 long-chain polyunsaturated fatty acids (LCPUFA) and high dose vitamin D in pregnancy, resulting in significantly reduced risk of asthma and wheeze in childhood^3–6^. The mechanisms for these effects are unknown. However, both have been shown to affect the developing immune system; n-3 LCPUFA compete with arachidonic acid-derived eicosanoids, modulate key regulatory cytokines, and is involved in the maturation of Th cells^7,8^, whereas vitamin D, and associated vitamin D receptors (VDRs), are involved in the maturation of regulatory T-cells (Tregs), the production of antimicrobial peptides, and the production of the tight junction proteins cadherins^9–11^. In addition to being immune regulatory, n-3 LCPUFA and vitamin D intake have been associated with gut microbial changes in both human observational studies and murine intervention models^12–14^.

In recent years the human microbiome has received increased attention as a potential contributor to disease development, especially the earliest microbial compositions^15^. In relation to asthma and allergic diseases, both early life bacterial composition in the gut^16–18^ and specific bacterial genera in the neonatal airways^19,20^ have been associated with increased disease risk later in life.

The aim of this study is to analyze if maternal supplementation with n-3 LCPUFA and vitamin D affects the microbiota of mother and child. Furthermore, we want to elucidate if the protective effect of these supplementations on asthma can be mediated by the microbiota. This is done using the Copenhagen Prospective Studies on Asthma in Childhood 2010 (COPSAC2010) mother-child cohort, consisting of 736 women and their children participating in a nested factorial randomized clinical trial of n-3 LCPUFA and vitamin D supplementation in pregnancy^21^. Microbiota samples were acquired from the pregnant women (vagina) and at regular intervals during infancy of the child (airways (hypopharyngeal aspirates), and feces) and are in this study analyzed with 16S rRNA gene amplicon sequencing.

In our study the dietary interventions affects the infant airways, but not the infant fecal or maternal vaginal microbiota. We observe changes in overall beta diversity, which in turn associates with a change in immune mediator profile. The interventions also affects the airway microbial maturation and the relative abundance of specific bacterial genera. Finally, using mediation analysis we observe that the changed airway microbiota is a minor and non-significant mediator of the protective effect of the dietary interventions on risk of asthma.

## Results

### Study population

Of the 736 women participating in the COPSAC2010 cohort, 693 were included in the n-3 LCPUFA trial and 580 in the vitamin D trial and delivered at least one microbial sample to our study. 695 children born to these women were included in the n-3 LCPUFA trial and 581 in the vitamin D trial and delivered at least one microbial sample. The same cohort was subject to both interventions in a factorial design with some mothers receiving only placebo, some mothers receiving either supplement, and some mothers receiving both. Randomized allocation among the included women produced comparable groups with respect to baseline characteristics (Table 1).

**Table 1:** Baseline characteristics of the n-3 LCPUFA and Vitamin D randomized controlled trials.

Category	Variable	Overall (*n* (%))	n-3 LCPUFA (*n* (%))	Placebo (*n* (%))	Vitamin D (*n* (%))	Placebo (*n* (%))
	*n*	693	344	349	294	286
Socioeconomics (maternal)	Age; mean (sd)	32.29 (4.36)	32.33 (4.27)	32.24 (4.46)	32.50 (4.33)	32.01 (4.32)
	Level of education:					
	Low	55 (7.9)	25 (7.3)	30 (8.6)	20 (6.8)	25 (8.7)
	Medium	446 (64.4)	221 (64.2)	225 (64.5)	185 (62.9)	189 (66.1)
	High	192 (27.7)	98 (28.5)	94 (26.9)	89 (30.3)	72 (25.2)
	Income level:					
	Low	67 (9.7)	33 (9.6)	34 (9.7)	27 (9.2)	27 (9.4)
	Medium	366 (52.9)	178 (51.9)	188 (53.9)	151 (51.4)	153 (53.5)
	High	259 (37.4)	132 (38.5)	127 (36.4)	116 (39.5)	106 (37.1)
During pregnancy	Smoking	54 (7.8)	21 (6.1)	33 (9.5)	20 (6.8)	26 (9.1)
	Cat or dog in home	234 (34.1)	117 (34.4)	117 (33.8)	88 (30.3)	105 (37.0)
	Antibiotic use	253 (36.6)	128 (37.3)	125 (35.8)	102 (34.8)	103 (36.0)
Child	Sex (male)	354 (51.1)	168 (48.8)	186 (53.3)	154 (52.4)	144 (50.3)
	Race (caucasian)	664 (95.8)	332 (96.5)	332 (95.1)	282 (95.9)	274 (95.8)
	Season of birth:					
	Winter	212 (30.6)	98 (28.5)	114 (32.7)	107 (36.4)	102 (35.7)
	Spring	185 (26.7)	94 (27.3)	91 (26.1)	53 (18.0)	53 (18.5)
	Summer	148 (21.4)	74 (21.5)	74 (21.2)	62 (21.1)	57 (19.9)
	Fall	148 (21.4)	78 (22.7)	70 (20.1)	72 (24.5)	74 (25.9)
	Duration of breastfeeding	245 (155)	249 (159)	240 (156)	243 (145)	247 (165)
Birth	Preterm delivery	27 (3.9)	12 (3.5)	15 (4.3)	10 (3.4)	9 (3.1)
	Nulliparity	318 (45.9)	153 (44.5)	165 (47.3)	121 (41.2)	140 (49.0)
	Antibiotics to mother	221 (32.0)	111 (32.4)	110 (31.7)	97 (33.2)	87 (30.5)
	Antibiotics to child	18 (2.6)	10 (2.9)	8 (2.3)	5 (1.7)	9 (3.2)
	Hospitalized	77 (11.1)	39 (11.3)	38 (10.9)	27 (9.2)	27 (9.5)
	Delivery mode:					
	Acute CS	84 (12.1)	47 (13.7)	37 (10.6)	39 (13.3)	33 (11.5)
	Vaginal	544 (78.5)	266 (77.3)	278 (79.7)	225 (76.5)	226 (79.0)
	Planned CS	65 (9.4)	31 (9.0)	34 (9.7)	30 (10.2)	27 (9.4)

Percentages or standard deviations in parenthesis. No significant allocation differences were observed (chi-sq or *t*-test, *p* < 0.05)

### Longitudinal development of the microbial community

**Fig. 1:**
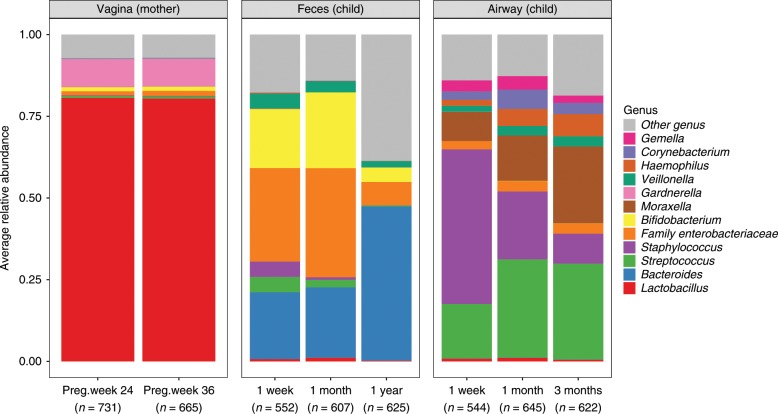
Bacterial community composition in children’s feces and airway, and maternal vaginal samples over time. The community composition is represented by the 12 most abundant genera. Each bar is represented by 544 to 665 samples. Source data are provided as a Source Data file.

We successfully sampled and sequenced the V4 region of the 16S bacterial rRNA gene in 4991 samples, with a mean sequencing depth of 54,682. The total number of unique OTUs found across all samples was 6846 (see Supplementary Table 1). Here follows a short resume of the microbial longitudinal development in the maternal vaginal, and infant fecal and airway samples of the COPASAC2010 mother-child cohort: In the maternal vaginal samples the average bacterial composition in samples from week 24 and 36 appeared similar and were dominated by the genera *Lactobacillus* and *Gardnerella* accounting for ~80% and 10% of the sequencing reads, respectively (Fig. 1).

In the infant fecal samples the composition in samples from the infants taken at 1-week and 1-month were dominated by *Bifidobacterium*, *Enterobacteriaceae*, and *Bacteroides*. In the 1-year fecal samples, we observed a significant decrease in *Enterobacteriaceae*, *Staphylococcus*, *Streptococcus* and *Bifidobacterium* in favor of *Bacteroides* and a generally larger proportion of overall lower-abundance genera, including *Faecalibacterium* and *Prevotella*.

In the infant airway samples the three major genera taken at 1-week after birth were *Staphylococcus*, *Streptococcus* and *Moraxella*. In the subsequent samples from 1-month and 3-months, we observed a gradual increase in the relative abundance of *Streptococcus*, *Moraxella*, and *Haemophilus*, as well as a decrease in *Staphylococcus*. More details on the individual sample types can be found in previous publications^16,22^ (Mortensen, M. S. et al. Stability of Vaginal microbiota during pregnancy and its importance for early infant microbiota. Unpublished)

### Effects of the n-3 LCPUFA and vitamin D interventions

The vaginal samples were taken at pregnancy week 36, twelve weeks into the supplementation with n-3 LCPUFA and vitamin D, totaling 665 samples, and did not show any significant differences in alpha diversity as a result of either intervention calculated using the Shannon index (see Supplementary Fig. 1). Similarly, no significant changes in beta diversity were observed in the vaginal samples as a result of the two dietary interventions (Table 2).

**Table 2:** The effects of n-3 LCPUFA and Vitamin D vs. placebo on microbial beta-diversity.

Compartment	Time point	n-3 LCPUFA F/R2/*P*-value	Vitamin D F/R2/*P*-value
Maternal vagina	Week 36	1.517/0.002/0.205	0.824/0.002/0.399
Child feces	1-week	0.172/0.001/0.969	0.350/0.002/0.955
	1-month	0.827/0.001/0.472	0.339/0.001/0.865
	1-year	1.270/0.002/0.250	0.277/0.001/0.973
Child airway	1-week	0.277/0.001/0.987	0.350/0.001/0.955
	1-month	**4.228/0.007/0.004**	**3.740/0.007/0.005**
	3-month	0.172/0.000/0.993	1.140/0.002/0.297

Effects were quantified with F statistics, R2, and *P*-values, as determined by PERMANOVA on weighted UniFrac distances. Significant *p*-values (*p* < 0.05) are shown in bold

The infant fecal microbiota was sampled at 1-week, 1-month, and 1-year after birth, totaling 552, 607, and 625 samples, respectively. No significant effects were seen of the two dietary interventions neither on alpha diversity (Supplementary Fig. 1) nor beta diversity (Table 2) at any of the time points.

**Fig. 2:**
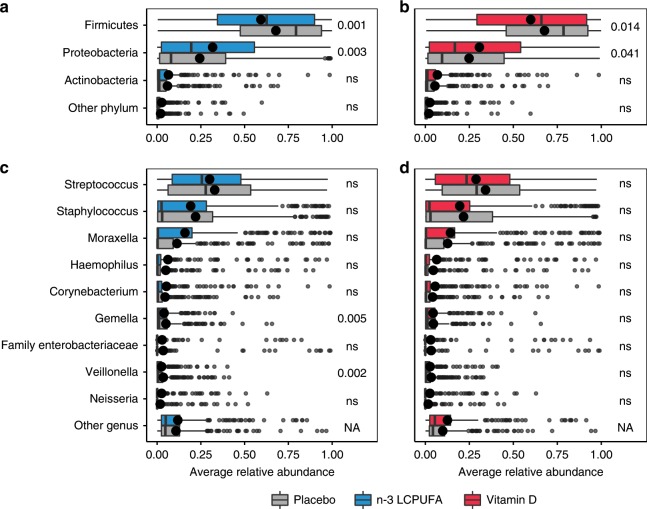
Relative bacterial abundances stratified by intervention groups for the 1-month airway samples. Relative abundances are shown at the phylum-level (**a** and **b**), and genus-level (**c** and **d**). The three most abundant phyla and nine most abundant genera are shown. Phylum level DA statistics were performed using Wilcoxon rank sum test and genus level DA using the metagenomeseq feature model. Boxplots with first and third quartiles corresponding to the lower and upper hinge, the median represented by a vertical line, the mean by a black dot, upper/lower whiskers extend to the largest/smallest value no further than 1.5 * inter-quartile range (IQR) from the hinge, and outliers are shown as gray circles. In bold are crude *p*-values reaching FDR corrected significance at level 0.1. *n* = 653 and 541 independent samples for the n-3 LCPUFA (**a** + **c**) and Vitamin D (**b** + **d**) plot, respectively. Source data are provided as a Source Data file.

Infant airway samples were taken at 1-week, 1-month, and 3-months, totaling 544, 645, and 622 samples, respectively. No effects were seen on alpha diversity at any of the three time points (see Supplementary Fig. 1). However, at 1-month of age, both interventions affected the microbial composition (PERMANOVA, n-3 LCPUFA (*F* = 3.74, *p* = 0.005), and Vitamin D (*F* = 4.228, *p* = 0.004) (Table 2). No interaction between the two interventions was observed (PERMANOVA, *F* = 0.49, *p* = 0.785), and the effect of each intervention remained significant after adjusting for common covariates, which might affect the microbiota (delivery, older siblings, antibiotics during pregnancy, antibiotics in the first month of life, birth season, cat at home at birth, dog at home at birth, and sex). To study the microbial composition effects at 1-month in more detail a differential abundance (DA) analysis was made on the most abundant phyla and genera (Fig. 2). Both interventions led to a significant decrease in Firmicutes and a corresponding increase in Proteobacteria (Fig. 2a, b). These changes seemed mainly to be driven by decreases in the genera *Streptococcus* and *Staphylococcus* with an increase in *Moraxella*, although neither of these individual changes were statistically significant (Fig. 2c, d). In addition, n-3 LCPUFA supplementation resulted in a significant decrease in the two genera *Gemella* and *Veillonella* (Fig. 2c). An additional DA analysis was performed on OTU level including also less abundant OTUs, hoping to find rare but important taxa affected by the interventions (Supplementary Fig. 2). n-3 LCPUFA significantly (metagenomeSeq, *p* < 0.05) increased the abundance of four OTUs, and decreased the abundance of six OTUs including three *Veillonella* (Supplementary Fig. 2, and Supplementary Table 2). Vitamin D increased the abundance of six OTUs including two *Neisseria* and two *Haemophilus* while decreasing the relative abundance of seven *Streptococcus* OTUs, five of which was putatively identified a *S. pneumoniae*, and the rest as *S. mitis* or *S. oralis*. However, none of the effects on single OTUs were significant after correcting for multiple testing (FDR, *p* < 0.1).

The 1-week and 3-month infant airway samples did not reveal any beta diversity differences between the intervention groups (Table 2).

### Additive effects of n-3 LCPUFA and Vitamin D

**Fig. 3:**
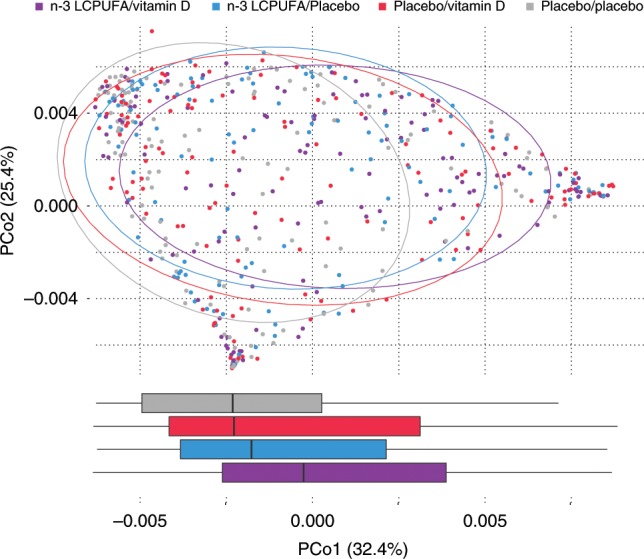
Ordination of 1-month airway samples stratified by intervention group. An additive effect is apparent in the right shift observed over PCo1 as samples are subject to either (n-3 LCPUFA/placebo (*n* = 125), or placebo/vitamin D (*n* = 141)) or both prenatal dietary interventions (n-3 LCPUFA/vitamin D (*n* = 131)) compared to double-placebo (placebo/placebo (*n* = 144)) (*p* < 0.001, Wilcoxon rank sum test). Boxplots represent the PCo1 values of each intervention group with first and third quartiles corresponding to the lower and upper hinge, the median represented by a vertical line, and the upper/lower whiskers extend to the largest/smallest value no further than 1.5 * inter-quartile range (IQR) from the hinge. Source data are provided as a Source Data file.

**Fig. 4:**
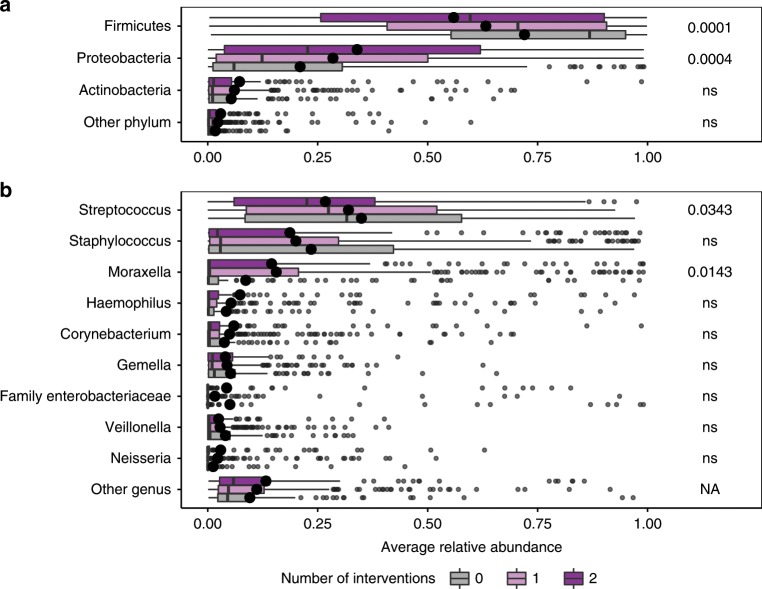
Additive effects of the interventions on the 1-month airway samples. Relative abundances are shown on (**a**) Phylum level and (**b**) Genus level. The correlation was analyzed by Spearman’s rank correlation coefficient and p-values below 0.05 are shown. The *p*-values in **b** are not significant when correcting for multiple testing using an FDR corrected significance of 0.1. Boxplots with first and third quartiles corresponding to the lower and upper hinge, the median represented by a vertical line, the mean by a black dot, upper/lower whiskers extend to the largest/smallest value no further than 1.5 * inter-quartile range (IQR) from the hinge, and outliers are shown as gray circles. *n* = 144, 266, and 131 for the zero, one, and two intervention groups, respectively. Source data are provided as a Source Data file.

Since both interventions showed significant effects on the 1-month airway microbial composition and similar shifts on both phylum and genus levels, the possibility of an additive effect was further explored using a PCoA stratified by the two intervention groups (Fig. 3). We observed a rightward shift on PCo1 according to intervention, i.e., with the group receiving both n-3 LCPUFA and vitamin D on the right, the groups receiving only one intervention in the center, and the group receiving placebo in both interventions on the left (*p* < 0.001, Wilcoxon rank sum test). Building on this concordance between n-3 LCPUFA and vitamin D effects, a correlation analysis was performed, using the number of interventions against the relative abundances of individual phyla and genera (Fig. 4). This confirmed the previous results of the individual interventions, showing a stepwise significant decrease in Firmicutes and an increase in Proteobacteria as a function of the number of interventions received (Fig. 4a). At the genus level, *Streptococcus* significantly decreased and *Moraxella* increased (Fig. 4b), although these exploratory findings did not reach FDR corrected significance at level 0.1.

Additive increases were also seen for *Neisseria*, *Corynebacterium*, and *Haemophilus* and additive decreases for *Staphylococcus*, *Gemella* and *Veillonella*, although neither of these trends were statistically significant (Spearman’s rank correlation coefficient, *p* > 0.05) (Fig. 4b). The grouping of the cohort into number of interventions (0, 1, and 2) still produced comparable groups with respect to baseline characteristics (Supplementary Table 3).

### Mediation analysis

After documenting the effect of n-3 LCPUFA and Vitamin D on the 1-month airway microbiota, we wanted to study if these changes could serve as a mediator for the previously observed protective effect of the interventions on childhood asthma. To do this we used PCo1 from Fig. 3 as a surrogate of the intervention induced microbiota changes in a mediation analysis using parametric survival regression models with the debut of persistent wheeze or asthma as end-points (Supplementary Table 4). We found that the 1-month airway microbiota effect from n-3 LCPUFA supplementation could account for an estimated 9% of the total asthma prevention effect till age 5 years (*p* = 0.11) previously published^3^. Similarly, 5.5% of the previously published vitamin D effect on persistent wheeze till 3 years of age could be mediated by changes in the 1-month airway microbiota (*p* = 0.39)^4^. However, none of the minor mediation effects were statistically significant, suggesting that the microbial changes observed as a result of the two dietary supplements had a minor or no effect on later risk of asthma.

### The interventions and gut and airway microbial maturation

As early microbial colonization patterns have previously been linked to later health outcomes we wanted to study the influence of the interventions on the early life microbial maturation. To do that we calculated the microbiota-by-age *z*-score (MAZ) for the gut and airway samples, with both compartments represented by three sampling time points^23^. No effect of either intervention on MAZ was seen on any of the gut samples, and on the 3-month airway samples. However, n-3 LCPUFA decreased the airway maturation in the 1-week time point (*t*-test, *p* = 0.004), and increased the maturation in the 1-month time point (*t*-test, *p* = 0.01). The Vitamin D intervention had a weaker and not significant effect, although the same trend was observed at both timepoints. An additive effect of the interventions was also observed decreasing the MAZ with 0.2 per intervention (*p* = 0.007) at the 1-week and increasing with 0.1 per intervention (*p* = 0.05) at 1-month.

### Interventions, airway microbiota, and airway immunology

**Fig. 5:**
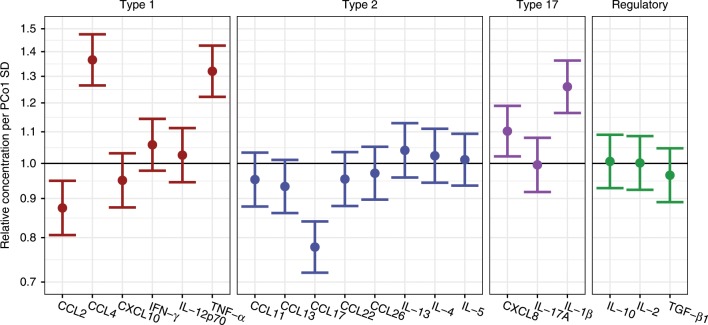
Associations between intervention driven microbiota changes in the 1-month airway and neonatal airway immunology. PCo1 from Fig. 3 was used as a metric for the intervention driven microbial changes and analyzed with the airway concentration of 20 local immune mediators in the nose, also measured at the 1-month time point. Linear models show that PCo1 is associated with several immune mediators, expressed as relative concentration ratios of immune mediators per standard deviation (SD) increase in PCo1, *n* = 585 independent samples. Error bars represent 95% confidence intervals. Source data are provided as a Source Data file.

To study the mechanism of the observed changes in airway microbiota in the 1-month time point, we analyzed the association between PCo1 from Fig. 3, affected by both interventions, and airway immunology taken at the same time point. We found a significant positive association between PCo1 and CCL4, TNF-α, CXCL8, and IL-1β, and a significant negative association between PCo1 and CCL2, and CCL17 (Fig. 5).

### Effect modulation by mode of delivery

To investigate possible mechanisms of the intervention effects on the 1-month airway microbiota, we examined potential effect modulation by mode of delivery. If the interventions had direct effects on the maternal gut microbiota, an assumed transfer to the child could be interrupted by cesarean section delivery. However, no significant interaction was observed neither for delivery mode on the global beta diversity variation (adonis, *p* = 0.58) nor the shift in PCo1 (linear model, *p* = 0.29) of the 1-month airway samples.

## Discussion

Third trimester supplementation with n-3 LCPUFA and high dose vitamin D had significant effects on the infant 1-month airway microbiota, with the interventions affecting the overall population structure and relative abundances of individual genera in a similar and additive manner. This suggests that both micronutrients share a common pathway modifying the infant airway microbiota. Alternatively, both lack of n-3 LCPUFA and vitamin D could be limiting factors of a general optimal fetal immune development, which would explain the observed parallel bacterial effects. Yet, such additive effects were not seen in our clinical end-points.

The effects of the interventions affected the relative abundance of several bacterial genera previously associated with inflammation, asthma and wheeze. The n-3 LCPUFA intervention resulted in a significant reduction in *Gemella* and *Veillonella* (Fig. 2), both positively associated with the development of asthma in a previous study of the same cohort^24^. Furthermore, in our study, a reduction in the genus *Streptococcus* was found to be significant in the additive model of the two interventions, and the abundance of seven *Streptococcus* OTUs both belonging to pathogenic and commensal species were significantly reduced as an effect of Vitamin D (Supplementary Fig. 2, and Supplementary Table 2). This genus has previously been associated with later asthma development when detected in the airways during early infancy^19,25^. *Streptococcus-*rich, *Gemella-*rich*,* and *Veillonella*-rich bacterial compositions have been shown to associate with an inflammatory phenotype in healthy adults, with increasing levels of Th17 cytokines including IL-1a and IL-1B^26^. In addition, Veillonella has been shown to correlate positively with a number of inflammatory cells and exhaled nitric oxide (eNO), further suggesting a causal relationship between a distinct airway microbiota, systemic and topical inflammation, and asthma pathogenesis^27^.

While we found significant effects on the 1-month airway microbiota, there were no effects on the 1-week and 3-month time points. The very dynamic initial colonization and development of the airway microbiota, as described previously, may in part explain this^22,28^. This was also apparent in the effect of the interventions on airway maturation, possibly influencing colonization progression in the dynamic period of our two earliest time points, but no longer detectable at the 3-month time point, shown in the litterature to be a more stable period in airway microbial development^29^. However, the airway MAZ index did not associate with later asthma risk, suggesting that the maturation effects might be a side effect of the interventions clinical effects instead of the cause. The speculated immune-priming effects of the interventions and the observed clinical effects on later asthma suggests that the observed mechanism could penetrate in a certain so-called “window of opportunity” in early infancy; a period where the developing immature immune system and the highly dynamic airway microbiota mutually influence each other^30^. This time-sensitivity has been observed in several other microbiome studies, although the precise demarcations of such windows are not yet clearly defined^16,18,23^. Interestingly, there were no detectable effects on the maternal vaginal microbiota in pregnancy, nor the early gut microbiota of the children, which for Vitamin D has been observed before in a similar high dose vitamin D cohort study^31^.

The mechanism whereby the interventions affected the airway bacteria of the neonates of our study is still unknown, although we observed strong associations between the airway microbiota composition most affected by the interventions and several immune mediators including proinflammatory TNF-α and IL-1β. This could suggest that a more active and stimulated young immune profile affected the colonization pattern of the early airways, which has been linked to a reduced risk of inflammatory diseases in later life^32,33^. However, the direction of causality could also be opposite, with specific bacteria leading to a properly stimulated immune maturation. Several other possible mechanisms can be found in the litterature, e.g., with n-3 LCPUFAs being known for their anti-inflammatory effects, and the modulation of eicosanoid mediator synthesis is the likely primary mode of action related to asthma pathogenesis^8,34^. n-3 LCPUFAs have also been linked to a range of other anti-inflammatory mechanisms including the production of resolvins and protectins, and the reduction of pro-inflammatory mediators and oxidative stress^35,36^. It is unclear whether the maternal supplement only worked through a fetal priming effect^37,38^, or if higher levels were still present after birth, directly affecting the child immune cells.

We have previously demonstrated that the vitamin D supplementation upregulated local immune mediators in the airways related to type 1, type 2, type 17, and regulatory immune paths, at the age of 1-month, which could influence the bacterial colonization dynamics^4^, but the changes in the airway microbiota, as demonstrated in this study, could also be interacting with the immune maturation as discussed previously. Another possible pathway is an increased production of the antimicrobial peptides cathelicidin and β-defensin by the bronchial epithelial cells, which is stimulated by the hormonally active form of vitamin D^39^.

Despite affecting airway bacteria previously associated with inflammatory diseases, our results suggest that only a minor part of the protective effect on asthma/persistent wheeze from the prenatal n-3 LCPUFA (9%) and high-dose vitamin D (5%) supplementations could be explained by the observed early life microbial alterations in the airways, and this effect is uncertain as none of the mediation effects reached statistical significance. However, we believe that this is mainly a power issue as the overall clinical effects of n-3 LCPUFA just reaches significance. Splitting this effect into mediated (via microbiota) and direct (not mediated by microbiota), renders significance hard to achieve, as the clinical effects of n-3 LCPUFA is probably working through many different mechanistic pathways. These could include changes in fetal development including lung organogenesis, immune maturation and modulation of DNA methylation states^38^.

The major advantage of this study is that the effects of n-3 LCPUFA and vitamin D on the maternal and infant microbiota were tested in a double blind randomized clinical trial, reducing the risk of possible confounding inherent to observational studies. Another strength of this study is the clinical surveillance of the COPSAC2010 cohort, including longitudinal sampling from 695 mother-child pairs. All study participants were investigated by in-house trained research pediatricians and medical personnel ensuring comparable measurements in regard to both microbiota sampling and clinical diagnosis of asthma and persistent wheeze. It is another strength that several biological compartments were sampled in this study, including maternal vaginal and infant gut and airways. It is a limitation that additional compartments, including the maternal gut before and after intervention and infant microbiota right after birth, were not sampled. This would have allowed us to detect a possible fecal bacterial transfer event during birth^40–43^. However, an intervention manipulated maternal bacterial transfer during delivery was not observed in this study as the intervention effects were not modified by cesarean section. In addition, closer airway sampling around the 1-month time point could have helped in pinpointing the exact period of the effect of the two interventions. This study was performed using 16S rRNA gene amplicon sequencing, characterizing the effects of the interventions on the bacterial community composition, which does not cover possible effects on fungi, parasites and viruses inhabiting the human body. In addition, the technique is insensitive to functional differences between communities, which could be explored further in future studies employing metagenomic sequencing^44^.

## Methods

### Experimental design

The study was embedded in the population-based COPSAC2010 prospective mother-child cohort of 736 women and their children followed from week 24 of pregnancy, with the primary clinical endpoint persistent wheeze or asthma, which was diagnosed according to a previously validated quantitative algorithm^3,4,21^.

### Microbial sampling

Maternal vaginal swabs were sampled at the research unit at week 24 and 36 of pregnancy^45^. The infant airway was sampled using hypopharyngeal aspirates obtained at ages 1-week, 1-month, and 3-months, using a soft suction catheter passed through the nose^19^. Infant fecal samples were collected 1-week, 1-month, and 1-year after birth, either at the research clinic or by the parents at home using detailed instructions. Each fecal sample was mixed on arrival with 10% vol/vol glycerol broth. All samples were stored at −80 °C until DNA extraction.

### Immunological sampling

Airway immunology was assessed 1-month after birth by measuring unstimulated levels of 20 cytokines and chemokines in airway mucosal lining fluid sampled by nasosorption^4,46–48^. Briefly, a synthetic absorptive matrix (fibrous hydroxylatedpolyester sheets, Accuwik Ultra (cat no. SPR0730), Pall Life Sciences, Portsmouth, Hampshire, UK) was inserted in both nostrils for 2 min and stored at −80 °C until protein extraction, which was done by immersing both filter strips in 300 μL Milliplex assay buffer (Millipore, Cat no. L-AB, Mass) containing 1 protease inhibitor tablet (Roche) per 25 mL buffer, and then transferred to the cup of a cellulose acetate tube filter (0.22 μm) placed in an Eppendorf tube (Spin X centrifuge tube filter, cat no. CLS8161, Sigma-Aldrich, St Louis, Mo). The tube was centrifuged for 5 min at 16,000×*g*, 4 °C. The obtained protein extract was stored immediately at −80 °C until determination of immune mediators (cytokines and chemokines) using high-sensitivity electrochemiluminescence multiplex assays (Meso Scale Discovery, Rockville, Maryland). The 20 cytokines and chemokines include interleukin (IL)-12p70, CXCL10 (interferon gamma-induced protein [IP]-10), interferon (IFN)-γ, tumor necrosis factor (TNF)-α, CCL4 (macrophage inflammatory protein [MIP]-1β), CCL2 (monocyte chemoattractant protein [MCP]-1), CCL13 (MCP-4), IL-4, IL-5, IL-13, CCL11 (eotaxin-1), CCL26 (eotaxin-3), CCL17 (thymus-regulated and activation-regulated chemokine [TARC]), CCL22 (macrophage-derived chemokine [MDC]), IL-17, IL-1β, CXCL8 (IL-8), transforming growth factor (TGF)-β1, IL-10, and IL-2.

### Ethics

The study was conducted in accordance with the guiding principles of the Declaration of Helsinki and was approved by the National Committee on Health Research Ethics (H-B-2008-093 and H-B-2009-014), the Danish Data Protection Agency (2015-41-3696), and Danish Health and Medicines Authority (2612-3959). Both parents gave oral and written informed consent before enrollment. The trials are registered with ClinicalTrials.gov, identifier: NCT00798226 and NCT00856947.

### Dietary supplements

The women were randomized 1:1 to a daily dose of 2.4 g of n−3 LCPUFA (55% EPA and 37% DHA) in triacylglycerol form (Incromega TG33/22, Croda Health Care) or placebo (olive oil, containing 72% n–9 oleic acid and 12% n−6 linoleic acid (Pharma-Tech A/S)), given from pregnancy week 24 to 1 week postpartum^3^. In addition, the women were randomized 1:1 to a daily dose of 2400 IU vitamin D3 supplementation or matching placebo tablets (Camette A/S) from pregnancy week 24 to 1 week postpartum^4^. All women were instructed to continue supplementation of 400 IU of vitamin D3 during pregnancy as recommended by the Danish National Board of Health; thus, the study is a dose comparison of 2800 IU/d vs. 400 IU/d of vitamin D3 supplementation. These two interventions were crossed in a factorial design, yielding four groups (n-3 LCPUFA/vitamin D, n-3 LCPUFA/placebo, placebo/vitamin D and placebo/placebo).

### DNA extraction, sequencing, and bioinformatics

The DNA was extracted using the Mobio PowerMAG soil DNA isolation kit on the epMotion 5075 robotic platform acccording to manufacturer’s protocol. Extracted DNA was amplified using a two-step PCR reaction with the 16S rRNA gene primers 515 F (5′-GTGCCAGCMGCCGCGGTAA-3′) and 806 R (5′-GGACTACHVGGGTWTCTAAT-3′) targeting the V4 region^27^. The PCR conditions for the first PCR-step were 2 min of denaturation at 94 °C, followed by 30 cycles of 20 s at 94 °C (denaturing), 30 s at 56 °C (annealing), and 40 s at 68 °C (elongation), with a final extension at 68 °C for 5 min. In the second PCR-step the sequencing primers and adapters were added using the PCR conditions as above but with only 15 cycles. Finished libraries were quantified, equimolary pooled and concentrated using using the DNA Clean & Concentrator−5 Kit (Zymo Research, Irvine, CA, USA) according to the manufacturer’s instructions. Sequencing was done on the Miseq platform using the v2 kit (paired-end 250 bp reads). Sequencing reads were de-multiplexed by the Miseq Controller Software, trimmed using biopieces^49^, checked for chimeras using usearch^50,51^ OTU clustered using UPARSE^52^, and classified using Mothur^53,16^.

### Statistical analysis

All data analyses were conducted using the statistical software R v3.3.0^54^. Samples with less than 2000 reads (*n* = 146, 7.5%) were omitted from the analysis. Sequencing and taxonomy data handling, taxonomical agglomeration, alpha diversity (analyzed using the Shannon Index) and beta diversity (analyzed using weighted UniFrac distances^55^) estimates were done using the R-package “phyloseq”^56^. Differences in beta diversity were tested using permutational multivariate analysis of variance (PERMANOVA)^57^ with 10,000 permutations and visualized with principal coordinates analysis (PCoA) plots. Differential abundance was tested with the Wilcoxon rank sum test on phylum level and with the specialized tool for DA analysis of sparse data, the metagenomeSeq feature-model^58^, on genus and OTU level using dietary supplement as predictor, after filtering away sparse and rare genera (≥10% presence, ≥0.01% mean relative abundance). Detailed taxonomic assignment of significant OTUs was attempted using Blastn^59^ against the default NCBI nr/nt database, and all named bacterial hits with highest % identity and % query coverage was reported. Multiple inferences were controlled using the False Discovery Rate Control by Benjamini-Hochberg and significance thresholds were reported at level 0.1 owing to the exploratory nature of the study. Additive effects of the two dietary supplements were analyzed using the Spearman correlation coefficient. The two factor factorial design was subsequently represented as a single factor with three levels (0: placebo/placebo, 1: placebo/vitamin D or n-3 LCPUFA/placebo and 2: n-3 LCPUFA/vitamin D) and compared with microbiota markers (phyla, genera and PCoA features) using Spearman’s correlation coefficient. Microbiota-by-age *z*-scores (MAZ) of the airway and gut were calculated using a random forest model^16,23,29^, using a 10-fold cross validation procedure with calculation of: microbial maturity (MM) = (predicted microbiota age − median microbiota age); and MAZ = (MM/SD) of predicted microbiota age as metrics. In order to estimate the extent of asthma reduction from the individual interventions that could be mediated via 1-month airway microbiota modulations, parametric survival regression models, modeling time to asthma onset were built using the intervention and the first PCoA component from weighted UniFrac distances as covariates. This was done using the R-package “Survival”^60^ and “mediation”^61^. As such, the (average) direct effect (ADE) reflects the part of the intervention effect on asthma which cannot be explained by microbiota alterations, whereas the (average) causal mediated effect (ACME), represents the part of the intervention effect on asthma caused by microbiota changes. The proportion of the ACME effect in relation to the total intervention effect, in a simple metric, reflects the proportion of the intervention clinical effect on asthma mediated through the airway microbiota. Immune mediator concentrations (1-month time point) were standardized, total-sum normalized per sample, log-transformed and z-scored before further analysis. Immune mediators were analyzed in relation to PCo1 from Fig. 3 using linear models.

### Reporting summary

Further information on research design is available in the Nature Research Reporting Summary linked to this article.

## Supplementary information

Supplementary Information

Reporting Summary

## Data Availability

Summary and feature-level data underlying Figs. 1–5, and Supp Figs. 1, 2 are provided as Source Data files. The 16S rRNA gene sequences are publicly available at Sequence Read Archive (SRA) [https://www.ncbi.nlm.nih.gov/sra/] with the accession numbers PRJNA340273, PRJNA417357, PRJNA576765, and PRJNA579012. All other data that supports the findings in this study, including clinical data, are available from the corresponding author upon reasonable request. Participant-level personally identifiable data are protected under the Danish Data Protection Act and European Regulation 2016/679 of the European Parliament and of the Council (GDPR) that prohibit distribution even in pseudo-anonymized form, but can be made available under a data transfer agreement as a collaboration effort.

## Source data

Source Data
