# Supplementary material for: Prenatal dietary supplements influence the infant airway microbiota in a randomized factorial clinical trial
Source: Nat Commun. 2020 Jan 22;11:426. doi: 10.1038/s41467-020-14308-x (PMC6976654; doi:10.1038/s41467-020-14308-x)
Supplement: Supplementary file 1 — Supplementary Information [file 41467_2020_14308_MOESM1_ESM.pdf]

Supplementary Information

Prenatal Dietary Supplements Influence the Infant Airway Microbiota in a Randomized Factorial Clinical Trial

Hjelmsø et al

Includes:

-Supplementary Fig. 1. Effect of fish oil (n-3 LCPUFA) and vitamin D on bacterial diversity

-Supplementary Fig 2. Effect of n-3 LCPUFA and Vitamin D prenatal dietary supplements on bacterial OTUs in the 1-month airway

-Supplementary Table 1. DNA amplicon sequencing summary of the individual sample types and timepoints.

-Supplementary Table 2. Blast results of reference sequences of significant OTUs from Fig S2

-Supplementary Table 3. Baseline characteristics of the nested COPSAC2010 n-3 LCPUFA and Vitamin D randomized controlled trials

-Supplementary Table 4. Mediation analysis details

Supplementary Figures

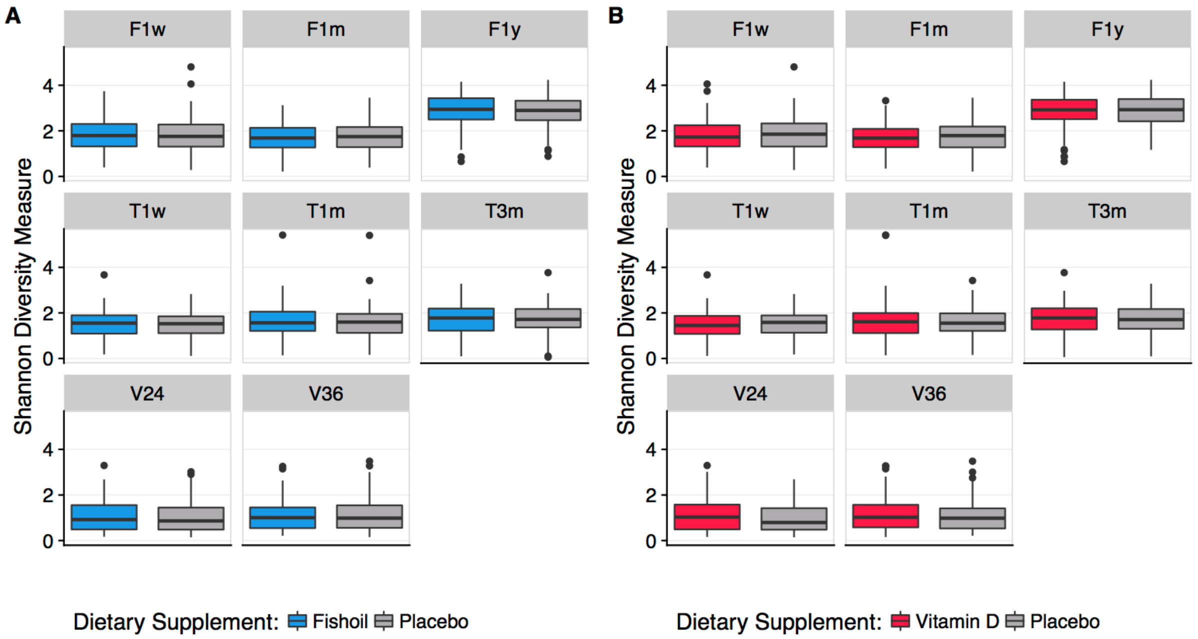

Supplementary Fig. 1. Effect of fish oil (n-3 LCPUFA) and vitamin D on bacterial diversity. Shannon diversity in the fecal (F), airway (T), and vagina (V) samples, taken at 1 week (1w), 1 month (1m), 1 year (1y), 24th pregnancy week (24), and 36th pregnancy week (36). No significant effect of either intervention was observed at any compartment/timepoint (Wilcoxon, p>0.05).

Boxplots with first and third quartiles corresponding to the lower and upper hinge, the median represented by a horizontal line, upper/lower whiskers extend to the largest/smallest value no further than 1.5 * inter-quartile range (IQR) from the hinge, and outliers are shown as black circles. n=see Supp Table 1. Source data are provided as a Source Data file.

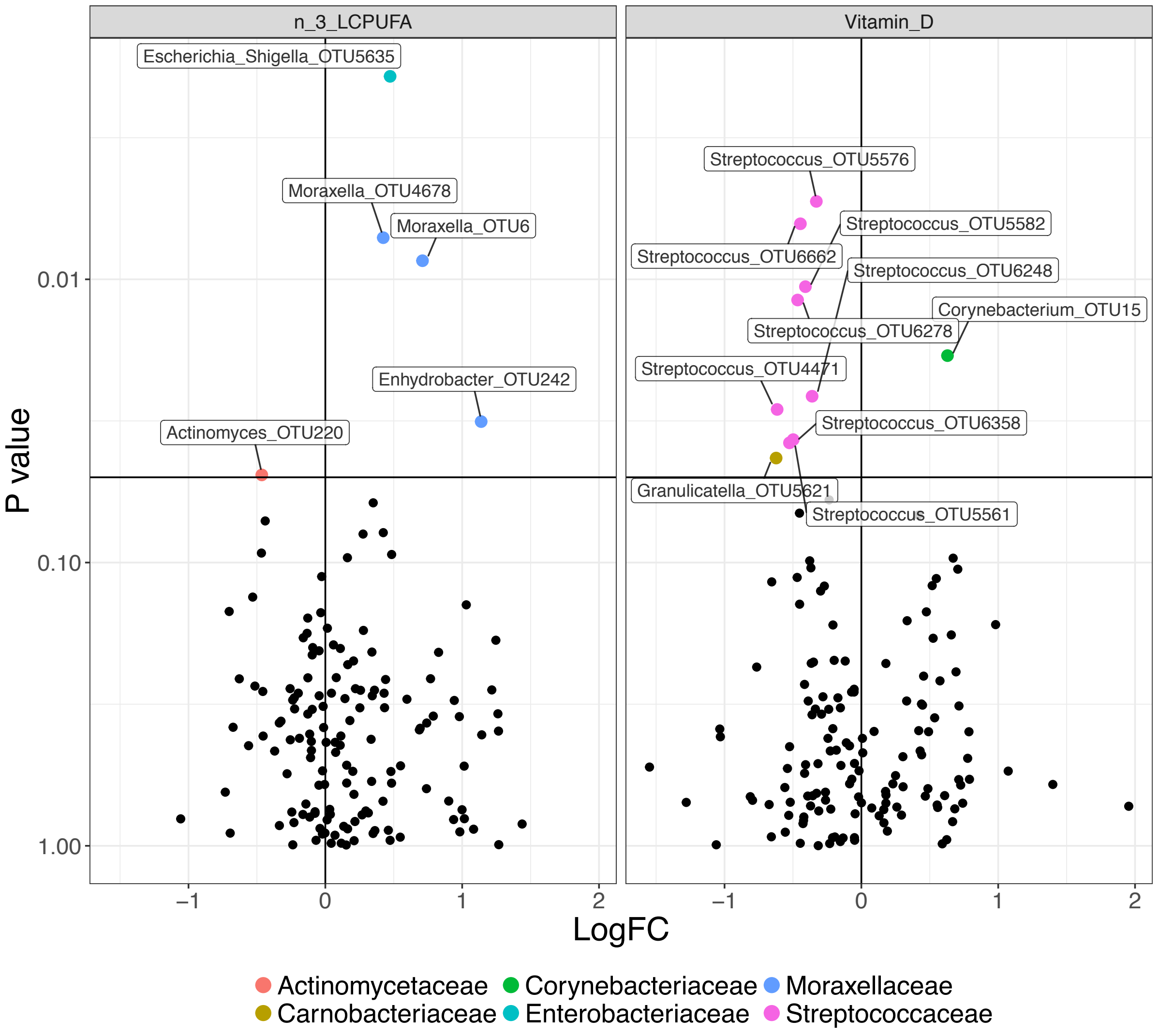

Supplementary Fig 2. Effect of n-3 LCPUFA and Vitamin D prenatal dietary supplements on bacterial OTUs in the 1-month airway. Vulcano plots show the log fold change (FC) of each OTU as an effect of each intervention on the x-axis and the p-value from a wilcoxon test of differences in the mean relative abundance on the y-axis. Horizontal line represent the $p<0.05$ cutoff. Significant OTUs are colored by taxonomic Family and labelled with OTU name. No OTUs remained significant efter correcting for multiple testing using a FDR threshold of 0.1. n=653 and 541 independent samples for the n-3 LCPUFA and Vitamin D plot, respectively. Source data are provided as a Source Data file.

Supplementary Tables

Sample type	Samples	OTUs	Mean read number	Median read number
Vagina week 24	731	1846	50325	47236
Vagina week 36	665	1788	51501	46996
Infant feces week 1	552	2791	63323	57495
Infant feces month 1	607	2450	57857	52122
Infant feces year 1	625	3271	58147	56962
Infant airway week 1	544	1627	49427	40824
Infant airway month 1	645	2649	58954	57527
Infant airway month 3	622	2098	49121	44875

Supplementary Table 1. DNA amplicon sequencing summary of the individual sample types and timepoints.

OTU Name	Species_name_from _Blast	Max score	Total score	Query cover	E value	Ident	Accession
Actinomyces_OTU220	Actinomyces graevenitzii strain PCH-184 16S ribosomal RNA gene, partial sequence	468	468	100%	5E-128	100.00%	MF099819.1
Corynebacterium_OTU15	Corynebacterium propinquum strain L0020-06F 16S ribosomal RNA gene, partial sequence	468	468	100%	5E-128	100.00%	MH447030.1
	Corynebacterium pseudodiphtheriticum strain V17 2012165 16S ribosomal RNA gene, partial se	468	468	100%	5E-128	100.00%	KF926062.1
Enhydrobacter_OTU242	Moraxella osloensis strain CFP312 16S ribosomal RNA gene, partial sequence	470	470	100%	1E-128	100.00%	MK283753.1
	Enhydrobacter aerosaccus strain BMC2N4_2 16S ribosomal RNA (16s ribosomal) gene, partial se	470	470	100%	1E-128	100.00%	MG996857.1
Escherichia_Shigella_OTU5635	Escherichia coli strain 382634_2f chromosome, complete genome	468	3246	97%	5E-128	98.49%	CP039403.1
	Uncultured Escherichia/Shigella sp. clone Otu055 16S ribosomal RNA gene, partial sequence	468	468	97%	5E-128	98.49%	MF039953.1
Granulicatella_OTU5621	Granulicatella elegans strain WHMH11 16S ribosomal RNA gene, partial sequence	516	516	94%	2E-142	98.63%	JN801174.1
Moraxella_OTU4678	Moraxella bovoculi strain 58086, complete genome	442	1769	100%	3E-120	98.05%	CP011381.2
	Moraxella lacunata strain PCH-094 16S ribosomal RNA gene, partial sequence	442	442	100%	3E-120	98.05%	MF073278.1
	Moraxella bovis strain CANIIIA 16S ribosomal RNA gene, partial sequence	442	442	100%	3E-120	98.05%	JN001954.1
Moraxella_OTU6	Moraxella catarrhalis strain MC8 chromosome	468	1873	100%	5E-128	100.00%	CP010902.1
	Moraxella nonliquefaciens strain 4663/62 16S ribosomal RNA, partial sequence	468	468	100%	5E-128	100.00%	NR_104938.1
Streptococcus_OTU4471	Streptococcus pneumoniae isolate GPS_ZA_821-sc-1950967 genome assembly, chromosome: 1	520	520	95%	2E-143	99.31%	LR536845.1
Streptococcus_OTU5561	Streptococcus pneumoniae isolate GPS_ZA_821-sc-1950967 genome assembly, chromosome: 1	520	520	96%	2E-143	98.97%	LR536845.1
Streptococcus_OTU5576	Streptococcus pneumoniae isolate GPS_ZA_821-sc-1950967 genome assembly, chromosome: 1	503	503	93%	2E-138	99.28%	LR536845.1
Streptococcus_OTU5582	Streptococcus mitis strain SK637 chromosome, complete genome	507	2028	96%	1E-139	98.28%	CP028415.1
	Streptococcus oralis strain 32P.4S2 16S ribosomal RNA gene, partial sequence	507	507	96%	1E-139	98.28%	MK131306.1
Streptococcus_OTU6248	Streptococcus mitis strain SK637 chromosome, complete genome	516	2065	96%	2E-142	99.65%	CP028415.1
	Streptococcus oralis strain 32P.4S2 16S ribosomal RNA gene, partial sequence	516	516	96%	2E-142	99.65%	MK131306.1
Streptococcus_OTU6278	Streptococcus mitis strain SK637 chromosome, complete genome	490	1961	92%	1E-134	97.25%	CP028415.1
	Streptococcus oralis strain 32P.4S2 16S ribosomal RNA gene, partial sequence	490	490	92%	1E-134	97.25%	MK131306.1
Streptococcus_OTU6358	Streptococcus australis clone WWE_MCE7_17 16S ribosomal RNA gene, partial sequence	514	514	93%	8E-142	98.63%	GU400321.1
Streptococcus_OTU6662	Streptococcus pneumoniae isolate GPS_ZA_821-sc-1950967 genome assembly, chromosome: 1	501	501	92%	6E-138	97.61%	LR536845.1

Supplementary Table 2. Blast results of reference sequences of significant OTUs from Fig S2. The top named hit was reported, and in case of several bacterial species with identical % ”query coverage” and % ”identity” all were reported. Blastn was used against the default NCBI nr/nt database.

Category	variable	0	1	2
	n	150	287	143
Socioeconomics (maternal)	Age	31.91 (4.51)	32.44 (4.22)	32.26 (4.37)
	Level of education:			
	Low	15 (10.0)	18 (6.3)	12 (8.4)
	Medium	96 (64.0)	194 (67.6)	84 (58.7)
	High	39 (26.0)	75 (26.1)	47 (32.9)
	Income level:			
	Low	14 (9.3)	27 (9.4)	13 (9.1)
	Medium	82 (54.7)	153 (53.3)	69 (48.3)
	High	54 (36.0)	107 (37.3)	61 (42.7)
During pregnancy	Cat or dog in home	61 (40.9)	87 (30.6)	45 (31.9)
	Antibiotic use	55 (36.7)	99 (34.5)	51 (35.9)
	Serum vitamin D	77.48 (25.49)	77.75 (26.45)	73.35 (22.76)
Child	Sex (male)	75 (50.0)	155 (54.0)	68 (47.6)
	Race (caucasian)	143 (95.3)	276 (96.2)	137 (95.8)
	Season of birth:			
	winter	58 (38.7)	98 (34.1)	53 (37.1)
	spring	26 (17.3)	58 (20.2)	22 (15.4)
	summer	31 (20.7)	58 (20.2)	30 (21.0)
	fall	35 (23.3)	73 (25.4)	38 (26.6)
	Duration of breastfeeding	245 (133)	246 (170)	239 (156)
Birth	Preterm delivery	6 (4.0)	8 (2.8)	5 (3.5)
	Nulliparity	78 (52.0)	120 (41.8)	63 (44.1)
	Antibiotics to mom	42 (28.0)	96 (33.8)	46 (32.2)
	Antibiotics to child	4 (2.7)	7 (2.5)	3 (2.1)
	Hospitalised	13 (8.7)	29 (10.1)	12 (8.4)
	Delivery:			
	acute CS	14 (9.3)	38 (13.2)	20 (14.0)
	vaginal	122 (81.3)	220 (76.7)	109 (76.2)
	planned CS	14 (9.3)	29 (10.1)	14 (9.8)

Supplementary Table 3. Baseline characteristics of the nested COPSAC2010 n-3 LCPUFA and Vitamin D randomized controlled trials. In this table grouped as zero (placebo/placebo), one (n-3 LCPUFA/placebo or placebo/Vitamin D), and two (n-3 LCPUFA/Vitamin D) interventions. Percentages or standard deviations in parenthesis. No significant allocation differences were observed (chi-sq or t-test, $p < 0.05$),

	Estimate	95% CI Lower	95% CI Upper	p-value	
n3-LCPUFA @ 5y					
ACME (average)	-417	-1250	52.22	0.090	
ADE (average)	-4160	-10900	-176.73	0.035	*
**Prop. Mediated (average)**	**0.09**	**-0.03**	**0.47**	**0.112**	
Vitamin D @ 3y					
ACME (average)	-37.7	-135	15.77	0.21	
ADE (average)	-438	-1420	237.63	0.27	
**Prop. Mediated (average)**	**0.0548**	**-0.719**	**0.88**	**0.39**	
Additive model @ 5y					
ACME (average)	227.33	-32.41	663.53	0.101	
ADE (average)	2870.95	459.88	5736.42	0.019	*
**Prop. Mediated (average)**	**0.0665**	**-0.0175**	**0.33**	**0.110**	

Supplementary Table 4. Mediation analysis details. In order to estimate the extent of asthma reduction from the individual interventions (n-3LCPUFA at 5 years and Vitamin D at 3 years) that could be mediated via 1-month airway microbiota modulations, parametric survival regression models, modeling time to asthma onset, were built using the intervention and the first PCoA component from weighted UniFrac distances as covariates. The (average) direct effect (ADE) reflects the part of the intervention which cannot be explained by microbiota alterations leading to differential frequency of asthma, and the (average) causal mediated effect (ACME), which is the intervention effect causing microbiota changes eventually leading to different frequency of asthma. The proportion of the ACME effect in relation to the total intervention effect, in a simple metric, reflects the proportion of the intervention clinical effect on asthma mediated through the airway microbiota.
