# Supplementary material for: Prenatal dietary supplements influence the infant airway microbiota in a randomized factorial clinical trial
Source: Nat Commun. 2020 Jan 22;11:426. doi: 10.1038/s41467-020-14308-x (PMC6976654; doi:10.1038/s41467-020-14308-x)
Supplement: Supplementary file 2 — Reporting Summary [file 41467_2020_14308_MOESM2_ESM.pdf]

nature research

Corresponding author(s): Hans Bisgaard
Last updated by author(s): Nov 27, 2019

Reporting Summary

Nature Research wishes to improve the reproducibility of the work that we publish. This form provides structure for consistency and transparency in reporting. For further information on Nature Research policies, see Authors & Referees and the Editorial Policy Checklist .

Statistics

For all statistical analyses, confirm that the following items are present in the figure legend, table legend, main text, or Methods section.

n/a	Confirmed	
☐	☒	The exact sample size (*n*) for each experimental group/condition, given as a discrete number and unit of measurement
☐	☒	A statement on whether measurements were taken from distinct samples or whether the same sample was measured repeatedly
☐	☒	The statistical test(s) used AND whether they are one- or two-sided<br>*Only common tests should be described solely by name; describe more complex techniques in the Methods section.*
☐	☒	A description of all covariates tested
☐	☒	A description of any assumptions or corrections, such as tests of normality and adjustment for multiple comparisons
☐	☒	A full description of the statistical parameters including central tendency (e.g. means) or other basic estimates (e.g. regression coefficient) AND variation (e.g. standard deviation) or associated estimates of uncertainty (e.g. confidence intervals)
☐	☒	For null hypothesis testing, the test statistic (e.g. *F*, *t*, *r*) with confidence intervals, effect sizes, degrees of freedom and *P* value noted<br>*Give P values as exact values whenever suitable.*
☒	☐	For Bayesian analysis, information on the choice of priors and Markov chain Monte Carlo settings
☒	☐	For hierarchical and complex designs, identification of the appropriate level for tests and full reporting of outcomes
☐	☒	Estimates of effect sizes (e.g. Cohen's *d*, Pearson's *r*), indicating how they were calculated

Our web collection on statistics for biologists contains articles on many of the points above.

Software and code

Policy information about availability of computer code

Data collection	Data was collected during the visits to the clinical research unit and stored in a dedicated custom online database. The data was double-checked against source data and subsequently locked. An audit trail was run routinely.
Data analysis	Fastq-files demultiplexed by the MiSeq Controller Software were trimmed for amplification primers, diversity spacers and sequencing adapters (biopieces), mate-paired and quality filtered (USEARCH v7.0.1090, parameter: -maxee 0.5). UPARSE was used for OTU clustering (>97% identity) as recommended, in particular removing singletons after de-replication. Chimera checking was performed with USEARCH against the Genomes OnLine Database as recommended. Representative sequences were classified (Mothur v1.25.0, using the wang function at 0.8 confidence threshold) against the Mothur formatted version of the Ribosomal Database Project v9. A phylogenetic tree was constructed from an alignment of representative sequences made with Mothur’s align_seqs function against the Greengenes database (may 2013 version). Alignments were then input to Fasttree in nucleotide mode.<br>All data analyses were conducted using the statistical software R v3.5.2, using the packages phyloseq, lme4, lmerTest, vegan, mixOmics, DMwR, mediation and ggplot2.

For manuscripts utilizing custom algorithms or software that are central to the research but not yet described in published literature, software must be made available to editors/reviewers. We strongly encourage code deposition in a community repository (e.g. GitHub). See the Nature Research guidelines for submitting code & software for further information.

Data

Policy information about availability of data

All manuscripts must include a data availability statement. This statement should provide the following information, where applicable:
- Accession codes, unique identifiers, or web links for publicly available datasets
- A list of figures that have associated raw data
- A description of any restrictions on data availability

Summary and feature-level data underlying Figs 1-5 and Supp Figs 1-2 are provided as Source Data files upon acceptance. The 16S rRNA gene sequences are

publicly available at Sequence Read Archive (SRA) [(https://www.ncbi.nlm.nih.gov/sra/]) with the accession numbers PRJNA340273, PRJNA417357, PRJNA576765 and PRJNA579012. All other data that supports the findings in this study, including clinical data, are available from the corresponding author upon reasonable request: Participant-level personally identifiable data are protected under the Danish Data Protection Act and European Regulation 2016/679 of the European Parliament and of the Council (GDPR) that prohibit distribution even in pseudo-anonymized form, but can be made available under a data transfer agreement as a collaboration effort.

Field-specific reporting

Please select the one below that is the best fit for your research. If you are not sure, read the appropriate sections before making your selection.

- [x] Life sciences
- [ ] Behavioural & social sciences
- [ ] Ecological, evolutionary & environmental sciences

For a reference copy of the document with all sections, see nature.com/documents/nr-reporting-summary-flat.pdf

Life sciences study design

All studies must disclose on these points even when the disclosure is negative.

Sample size	700 mothers were recruited to the COPSAC2020 cohort. 693 and 580 mothers participated in the clinical trial of the nested prenatal supplementation with n-3 LCPUFA and Vitamin D, respectively. This number was deemed sufficient for determining clinical effects of the interventions and also large enough to detect microbial changes in different compartments of the pregnant mothers and their children during early life.
Data exclusions	samples with a 16S rRNA amplicon sequencing depth of below 2000 reads were excluded as they were deemed unreliable and defined as failed.
Replication	To replicate the findings a new clinical trial would have to be done, including regular microbial sampling. We are working on financing such a study, but as of yet we are the only cohort with this experimental setup.
Randomization	The mothers were randomly allocated to either n-3 LCPUFA/placebo and Vitamin D/placebo. Due to the high number of mothers we were successful in creating two groups with similar baseline characteristics, and no significant allocation differences were observed (see Table 1) .
Blinding	Both investigators and study participants (Cohort mothers and children) were blinded during the study period.

Reporting for specific materials, systems and methods

We require information from authors about some types of materials, experimental systems and methods used in many studies. Here, indicate whether each material, system or method listed is relevant to your study. If you are not sure if a list item applies to your research, read the appropriate section before selecting a response.

Materials & experimental systems

n/a	Involved in the study	
[x]	[ ]	Antibodies
[x]	[ ]	Eukaryotic cell lines
[x]	[ ]	Palaeontology
[x]	[ ]	Animals and other organisms
[ ]	[x]	Human research participants
[ ]	[x]	Clinical data

Methods

n/a	Involved in the study	
[x]	[ ]	ChIP-seq
[x]	[ ]	Flow cytometry
[x]	[ ]	MRI-based neuroimaging

Human research participants

Policy information about studies involving human research participants

Population characteristics	The COPSAC2010 mother-child cohort consist of participants (pregnant women and their children) recruited in Denmark on the Island of Zealand. It is an unselected cohort thus reasonably reflecting mothers and children in the general Danish population.
Recruitment	Pregnant mothers were mailed and asked to participate in the prenatal supplementation trial with n-3 LCPUFA and Vitamin D, with the primary outcome of protecting against childhood Asthma. When the study participants were compared to pregnant mothers who did not want to participate there was a higher degree of study participants with asthma, exzema and hay fever. We do not believe this affected the effect of the prenatal supplements and the composition of the microbial samples from the mother and child.
Ethics oversight	the Danish National Committee on Health Research Ethics, the Danish Data Protection Agency, and the Danish Health and Medicines Authority

Note that full information on the approval of the study protocol must also be provided in the manuscript.

Clinical data

Policy information about clinical studies

All manuscripts should comply with the ICMJEguidelines for publication of clinical research and a completedCONSORT checklist must be included with all submissions.

Clinical trial registration	NCT00798226 and NCT00856947
Study protocol	The study protocol can be found in the paper "Deep phenotyping of the unselected COPSAC2010 birth cohort study" doi: 10.1111/cea.12213
Data collection	The mothers were seen regularly in the COPSAC clinic at Gentofte and satelite clinic in Næstved. Scheduled visits were planned at 1 week, 1, 3, 6, 12, 18, 24, 30, and 36 months, and regularly thereafter until age 7.
Outcomes	Asthma was the primary outcome, and eczema, allergic rhinitis and respiratory infections were the secondary outcomes. The diseases were diagnosed and treated by the doctors in the research clinic according to predefined algorithms. Daily diary cards were used from birth to monitor significant troublesome lung symptoms as previously analysed in detail including components of cough, wheeze, and dyspnoea, and use of b2-agonists, inhaled corticosteroids, and montelukast. Skin symptoms were monitored as active eczema and use of topical steroids. In addition, the diary cards monitored infections, categorized into common cold, pneumonia, pharyngitis, otitis, fever, gastrointestinal infection, and absence from day care institution because of illness. The diary cards were reviewed with the family by the research MD at each visit to validate symptom definitions. All information were subsequently entered into the online database and double-checked.<br>The bacterial community composition, as studied in this paper, was not part of either the primary or secondary outcomes. Thus this study is an exploratory one and should be interpreted as such.
